# Exploring the ecosystem of K-12 online learning: an empirical study of impact mechanisms in the post-pandemic era

**DOI:** 10.3389/fpsyg.2023.1241477

**Published:** 2023-09-27

**Authors:** Ping Wang, Feiye Wang, Zhiyuan Li

**Affiliations:** Faculty of Education, East China Normal University, Shanghai, China

**Keywords:** online learning, ecosystem theory, learning outcomes, impact mechanism, SEM model

## Abstract

With the normalization of the COVID-19 epidemic, online learning has gained prominence in the post-epidemic era. Gaining a comprehensive understanding of how online learning influences learning outcomes is essential for the success of large-scale online education initiatives. This study proposed a conceptual model based on an ecosystem theory to investigate the impact of K-12 online learning on academic achievement. A survey was conducted among 1,625 K-12 school students in Shenzhen, China, utilizing Structural Equation Modeling (SEM) for data analysis. The results indicate the following: (1) online learning engagement directly predicts academic achievement and mediates personal and environmental factors; (2) Family involvement and school support have similar impacts, with family involvement slightly stronger; (3) The Big Five Personality, especially conscientiousness, openness, and emotional stability, mediate the influence of family and school investment; and (4) School support affects academic achievement through online learning engagement, with emotional engagement being most significant. Our model illuminates the mediating role of online learning engagement, the impact of family involvement and school support, and the significance of Big Five Personality traits in K-12 online learning. This study contributes to the theoretical and practical understanding of the online learning ecosystem in the post-pandemic era, seldom explored in K-12 settings.

## 1. Introduction

Owing to technological advancements and the benefits it offers in terms of convenience and cost (Welsh et al., 2003; Ally, 2004), online learning has experienced substantial growth over the past two decades. This growth has been marked by market expansion (Need for LMS in HEO Driving Market Growth, 2018) and recognition from authoritative official institutions (Yeld, 2016). In recent years, the global spread of COVID-19 has led to strict social health measures, including school closures (Dreesen et al., 2020), further accelerating the prosperity of online learning (Liang et al., 2020). As a result, the significance and widespread application of online learning has been further reinforced in the post-pandemic era, garnering widespread attention from researchers in the community. Online learning presents both opportunities and challenges in the educational field after the COVID-19 pandemic (Greenhow et al., 2022).

Amid the surging tide of inquiries into online learning, a significant portion of research has gravitated toward adult college students. These inquiries have yielded insights spanning diverse domains, encompassing the design of learning activities (Rapanta et al., 2020), the identification of sustainable online learning factors (Chu et al., 2021), and the integration of interactive technologies to augment the learning experience (Ayu, 2020). Nevertheless, a conspicuous void persists in our understanding of the experiences of K-12 school students— a group of paramount significance in the post-pandemic educational landscape. Our research takes a purposeful stride in bridging this gap by delving into the intricate interplay connecting online learning and K-12 students’ learning outcomes.

Furthermore, the current academic discourse has indeed delved into the mechanisms through which online learning reverberates across educational outcomes. This exploration has encompassed dimensions such as self-directed learning approaches and attitudes (Shao et al., 2022), the influence of online learning technology (Gupta and Yadav, 2023), and the modes of teaching delivery (DeArmond et al., 2023). Although each of the aforementioned studies has delved into the impact mechanism from its respective perspective on singular and individual factors, to our knowledge, a systematic approach is lacking, leaving the underlying processes in a “black box.” Consequently, interactions among factors, mediation effects, and complex influence pathways remain understudied and undisclosed.

The Ecosystem Theory, proposed by Bronfenbrenner (2005), serves as a developmental psychology framework. It emphasizes intricate, reciprocal interactions between individuals and their surroundings. Increasing empirical evidence supports the notion that education operates as an organic, complex, and interconnected ecosystem, wherein student development outcomes are influenced by the interaction between individuals and their environment (Smith, 2013; Jelas et al., 2016). Drawing inspiration from the ecosystem theory, this paper aims to explore the factors that impact students’ learning outcomes in online learning by conducting empirical research. Thus, the research constructs a conceptual model of the online education ecosystem rooted in the ecosystem theory. Structural Equation Modeling (SEM) is employed to validate the model, utilizing data collected from K-12 students engaged in online learning in China. Our research endeavors encompass two main objectives. Firstly, it endeavors to decipher the complex interactions interweaving environmental and individual-level factors within the online learning ecosystem. Secondly, our study delves into personal factors at the individual level and the interplay between these personal factors and environmental influences.

## 2. Theoretical foundations

### 2.1. Online learning

Online learning encompasses various forms of education and training services delivered through Internet technology and platforms. It includes distance learning, e-learning, and other similar approaches. The terms “online learning” and “network learning” are often used interchangeably and are synonymous with “e-learning” and “remote learning” in English. This educational model stands in contrast to traditional face-to-face learning. Initially rooted in the field of distance learning, online learning primarily catered to adult learners and corporate training, enabling them to pursue studies remotely through network and information technology. However, with the advancement of digital technology, online learning has expanded its reach to encompass school education, becoming an indispensable and significant learning method for students (Harris, 2004).

### 2.2. Learning outcomes

Assessing the quality of online learning requires a careful examination of learning outcomes. Learning outcomes are statements that define what learners should know, understand, or demonstrate upon completing a learning process (Adam, 2004). It is crucial to focus on learners’ achievements rather than solely relying on teacher expectations, as highlighted by Kennedy (2006). Measuring learning outcomes offers numerous benefits to stakeholders such as students, teachers, and academic advisors, facilitating the optimization of the learning experience (Mahajan and Singh, 2017). However, in the realm of large-scale online learning, the concept of learning outcomes lacks uniformity among students. Cognitive and emotional variables are often employed as indicators to evaluate the overall quality of distance online learning projects (Paechter et al., 2010). Among cognitive variables, the paramount significance of academic performance is unmistakable (Lim et al., 2006). Thus, acknowledging its centrality and objectivity, this study positions academic performance as a faithful reflection of learning outcomes.

### 2.3. Ecosystem theory

Central to our theoretical framework is the concept of ecosystem theory, introduced by psychologist Urie Bronfenbrenner (Bronfenbrenner and Morris, 2007). This paradigm posits that individuals are not isolated entities but rather are sculpted by their interactive environmental milieu. Human development unfolds as an ongoing consequence of the dynamic interplay between individuals and their immediate surroundings. The ripple effect of distal factors reverberates through proximal factors, ultimately shaping the contours of individual development (Bronfenbrenner and Ceci, 1994).

**Figure 1:**
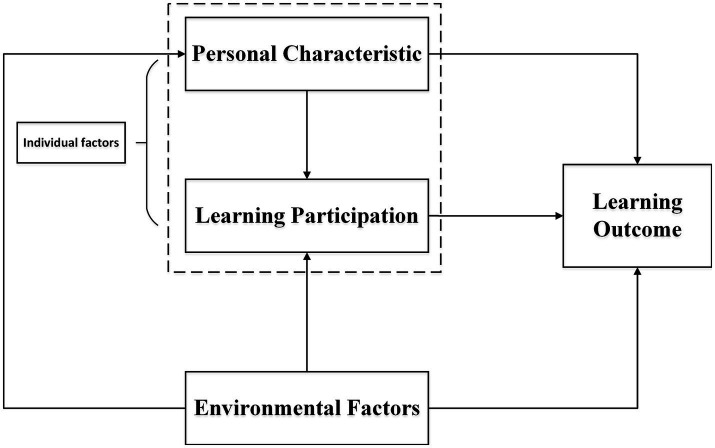
Conceptual model.

The examination of learning influence mechanisms has been partially shaped by the ecosystem theory. Scholars generally categorize factors affecting learning outcomes into two groups—individual factors and environmental factors. Environmental factors encompass social and cultural elements like family, schools, and communities (Chen, 2005). Individual factors refer to personal traits and performants such as student characteristics (Furnham et al., 2003), learning participation (Lei et al., 2018), and so on. Additionally, within individual factors, individual student characteristics (such as psychological processes) often play a crucial mediating role in interactions between external factors and other personal aspects (Jelas et al., 2016). Research by Li et al. (2010) discovered that students’ personal characteristics impact their learning participation, including perceptions of learning, investment in behavior, emotional engagement, and self-efficacy (Figure 1).

**Figure 2:**
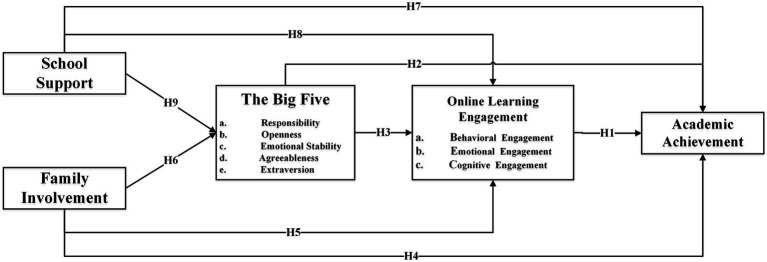
Conceptual roadmap.

Based on prior research, this study’s construction of a comprehensive model for the online learning ecosystem entails integrating environmental factors, personal characteristics, learning participation, and learning outcomes. The model depicted in Figure 2 is presented as a foundational representation that highlights the interactive essence of environmental factors, personal attributes, and process factors. These elements collectively exert influence on learning outcomes. It’s important to note that this visualization simplifies the intricate nested nature intrinsic to ecological systems, and we acknowledge that the actual interactions could be more complex. Moreover, we recognize that this model is one of many possible representations, and its purpose is to offer a conceptual roadmap that guides our investigation. In light of this, we clarify that the framework is a “conceptual model” rather than a “comprehensive theoretical framework.” We appreciate the evolving nature of this field and acknowledge that our conceptual model captures salient factors while recognizing the potential for refinement and expansion as our understanding evolves.

## 3. Literature review and hypotheses development

### 3.1. Individual factors

According to the conceptual model established in this study, both learning participation and personal characteristics fall under the category of individual factors.

#### 3.1.1. Learning participation

In traditional learning, the roles of teachers and families are crucial in facilitating learning. However, the advent of online learning has transformed this dynamic. Online learning grants learners greater autonomy and flexibility, but it also demands a higher level of engagement from them. Learning participation, also referred to as learning engagement, pertains to the extent of effort students invest in learning, as well as their interest and connection to the courses (Axelson and Flick, 2010). Fredricks et al. (2004) categorized three distinct forms of learning engagement: behavioral engagement, which involves active participation in courses and fulfilling course requirements; cognitive engagement, which encompasses the formulation of self-regulated learning strategies; and emotional engagement, which involves cultivating positive or negative affective states toward learning. In this study, online learning engagement serves as a representation of the learning participation process, with behavioral, cognitive, and emotional engagement serving as three specific dimensions of online learning engagement.

Several studies have highlighted the significance of learning engagement in predicting online learning outcomes. Lei’s research team (2018) conducted a meta-analysis of 69 independent studies and found a positive correlation between learning engagement and academic achievement. They discovered that all three types of learning engagement—behavioral, cognitive, and emotional—were positively associated with academic achievement. Building upon this, the present study proposes Research Hypothesis 1:

*H1*. Online learning engagement has a significant positive impact on academic achievement.

Furthermore, when considering the various forms of learning engagement that influence academic achievement, it is reasonable to hypothesize that these specific subsets within online learning engagement can also positively impact academic achievement. Therefore, we propose the following hypothesis:

*H1a, b, c*. Behavioral engagement, Emotional engagement, and Cognitive engagement in online learning engagement has a significant positive impact on academic achievement.

#### 3.1.2. Personal characteristics

With the popularity of online learning, researchers have turned their attention to individual differences and the specific needs of online learners. On a personal level, factors such as motivation and self-efficacy (Peng and Fu, 2021; Rorimpandey and Midun, 2021) have been acknowledged as influential contributors to learning outcomes. However, for a comprehensive understanding of the multifaceted nature of student development in the online learning environment, we have opted to incorporate the Big Five Personality traits as potential mediators. The Big Five personality model has gained prominence for its comprehensive and cross-cultural understanding of personality traits (De Raad, 2000). While motivation and self-efficacy are undoubtedly relevant, the inclusion of personality traits provides an additional layer of insight into the complexity of online learning outcomes. Costa and McCrae (1992) identified the five factors of the Big Five model as Extraversion, Agreeableness, Conscientiousness, Neuroticism, and Openness. These factors represent distinct aspects of an individual’s personality: sociability and energy in social interactions, interest in and empathy for others, organizational skills and self-discipline, emotional stability and response to stress, and receptiveness to new ideas and experiences. This study adopts the Big Five personality model to represent personal characteristics.

Studies examining the application of the Big Five personality traits in education have revealed their relationship with learning outcomes. Longitudinal research with British university students (Furnham et al., 2003) found that the Big Five traits are more important than cognitive ability, intellectual beliefs, and gender in predicting academic achievement. Conscientiousness positively correlates with academic achievement, while extraversion negatively correlates. Meta-analysis across different educational levels (Poropat, 2009) indicates significant associations between academic achievement and agreeableness, conscientiousness, and openness.

The Big Five personality traits of learners not only predict learning outcomes but also influence students’ learning participation. De Feyter et al. (2012) conducted analyses on longitudinal data from 375 Belgian college students, revealing that conscientiousness indirectly impacts academic achievement through learning motivation, and neuroticism positively affects academic achievement for students with higher self-efficacy. In a study by Quigley et al. (2022), extraversion predicted participation and performance, neuroticism predicted participation skills, emotional engagement, and performance, and agreeableness and openness predicted engagement and emotional engagement, respectively.

Through an extensive review of relevant literature, it becomes evident that there is a correlation between the Big Five personality dimensions and the learning outcomes and learning participation in online learning. This relationship has been widely discussed and examined in previous research. To ensure consistency in the impact direction of the theoretical framework, some scholars have reversed the interpretation of neuroticism within the Big Five personality traits, referring to it as emotional stability (Meng et al., 2021). Considering the aforementioned factors and taking into account the different facets of the Big Five personality traits, it is logical to posit that the Big Five, along with their specific subcategories, can have a positive influence on both academic performance and online learning engagement. Consequently, we present the following hypothesis:

*H2*. The Big Five has a significant positive impact on academic achievement.

*H2a, b, c, d, e*. Responsibility, openness, emotional stability, agreeableness, and extraversion have a significant positive impact on academic achievement.

*H3*. The Big Five has a significant positive impact on online learning engagement.

*H3a, b, c, d, e*. Responsibility, openness, emotional stability, agreeableness, and extraversion have a significant positive impact on online learning engagement.

### 3.2. Environmental factors

This study defines environmental factors as encompassing both family environment and school environment factors. While other environmental factors, such as the social environment, are undoubtedly relevant to online learning, the reduction of physical interaction in the online learning process leads to a decrease in the frequency of interactions among students and these environmental factors. In this context, we emphasize that the influence of family and school environments holds particular significance.

#### 3.2.1. Family environment

With the rapid development of internet technology, online learning has become mainstream. The COVID-19 pandemic has led to the adoption of online learning at home, turning formal learning into distance learning (Garad et al., 2021). Home-based online learning transforms the learning approach from group-oriented face-to-face instruction to self-directed learning with technological support. In this novel instructional model, the family plays a significant role as an immediate learning environment. Teachers provide remote supervision, while parents closely monitor and support their children’s learning (Wang and Yang, 2020). The growth of large-scale online learning has emphasized the importance of family involvement in education. Hence, this study considers family involvement as an indicator of the family environment.

On the one hand, scholars, especially Chinese research teams, have made progress in researching the relationship between family education involvement and students’ online learning outcomes. It has been found that family education involvement, including academic counseling, creating a conducive learning environment, and resource allocation, significantly predicts students’ online learning outcomes (Zhao et al., 2022). For instance, Bai’s research team (Bai et al., 2021) conducted an analysis based on 1,440 family questionnaires during the period of “suspended classes and non-stop learning” to examine the impact of family support factors on primary school students’ home learning outcomes. They found a significant positive correlation between family education involvement and home learning outcomes.

On the other hand, online learning presents unique challenges that require students to demonstrate self-management and self-motivation. Home learning support plays a crucial role in influencing students’ learning engagement, as family involvement can significantly predict students’ level of engagement. A longitudinal study focusing on education revealed that parents’ educational expectations for their children and home-school interaction positively impact various aspects of students’ learning engagement, including learning participation, self-efficacy, and intrinsic motivation (Fan and Williams, 2010). Similarly, in the context of online learning, scholars have observed that high parental involvement among elementary and junior high school students helps alleviate academic and emotional burnout symptoms (Zhao et al., 2022).

Furthermore, numerous studies have highlighted the substantial positive influence of family education involvement on students’ learning outcomes. However, this relationship is not a simple linear one, as it encompasses various factors, including students’ personal characteristics. Steinmayr et al. (2010) examined the mediating role of children’s intelligence and personality in the relationship between family background and children’s learning. Their findings revealed that certain personality traits, such as openness and conscientiousness, partially mediate the connection between parents’ educational background and children’s academic achievement. Notably, even after controlling for children’s intelligence, the mediating effect of personality traits persisted.

Based on the analysis of relevant literature, it is evident that there exists a certain correlation between family involvement with learning outcomes, learning engagement, and the Big Five personality traits. However, further research and discussion are needed to explore the specific nature of this relationship, the degree of correlation, and the underlying mechanisms. Also considering the specific forms of the Big Five and learning engagement, this study proposes research hypotheses 4, 5, and 6, which are as follows:

*H4*. Family involvement has a significant positive impact on academic achievement.

*H5*. Family involvement has a significant positive impact on online learning engagement.

*H5a, b, c*. Family involvement has a significant positive impact on behavioral engagement, emotional engagement, and cognitive engagement.

*H6*. Family involvement has a significant positive impact on the Big Five.

*H6a, b, c, d, e*. Family involvement has a significant positive impact on responsibility, openness, emotional stability, agreeableness, and extraversion.

#### 3.2.2. School environment

Online teaching has revolutionized the learning experience for students; however, it has not altered the fundamental relationship between teaching and learning (Stone and Springer, 2019). Despite the shift to online learning, effective teaching practices and learner engagement remain critical for achieving meaningful learning outcomes (Hodges et al., 2020). Schools continue to serve as the cornerstone of education. Within the realm of online learning, student learning outcomes are influenced by various factors associated with school support, including the quality of teachers, principal leadership, and the utilization of instructional platforms and tools (Rahman, 2021; Zhou et al., 2022). This study adopts school support as a measure of the school environment.

School support directly influences students’ learning outcomes. In an online learning study conducted by Baber (2020) among undergraduate students during the COVID-19 pandemic, it was discovered that classroom interaction, student motivation, course structure, teacher knowledge, and facilitation all positively influenced students’ perceived learning outcomes and satisfaction. Additionally, Cai (2021) developed a structural equation model based on the control-value theory and found that teachers’ preparation for online teaching significantly predicted the effectiveness of online learning among undergraduate students.

School support can influence students’ learning outcomes by affecting their learning engagement. Wan et al. (2021) conducted a quantitative study involving 4,841 Chinese college students, which revealed that perceived teacher support and online learning platform experience have an impact on college students’ online learning engagement. Similarly, Guo and Hu (2021) conducted a questionnaire survey with 635 Chinese college students to investigate the relationship between teachers’ behavior, students’ learning engagement, and learning outcomes. The findings indicated that learning engagement in online teaching partially mediates the relationship between teachers’ caring behavior and learning satisfaction.

Behavioral genetics research has demonstrated that both genes and the environment contribute to individual variations in personality traits among children and adolescents (Heiman et al., 2004). Considering the school environment as one of the environmental factors, it should play a role similar to family input in shaping academic learning outcomes through the joint effect with students’ personality characteristics. For instance, in a study conducted by Gina (Pancorbo et al., 2022) with middle school students, it was found that when teachers exhibit similar character traits to students, they are more likely to be liked by students and foster active interactive learning.

Based on the relevant literature, a correlation has been observed between school support and learning outcomes, learning engagement, and the Big Five personality traits. Referring to the research hypothesis on family involvement, this study presents research hypotheses regarding school support:

*H7*. School support has a significant positive impact on academic achievement.

*H8*. School support has a significant positive impact on online learning engagement.

*H8a, b, c*. School support has a significant positive impact on behavioral engagement, emotional engagement, and cognitive engagement.

*H9*. School support has a significant positive impact on the Big Five.

*H9a, b, c, d, e*. School support has a significant positive impact on responsibility, openness, emotional stability, agreeableness, and extraversion.

In summary, based on the established research hypotheses, this study has developed a conceptual model, as depicted in Figure 2.

## 4. Method

### 4.1. Procedure and participants

Most of the current empirical research on large-scale online education encounters several issues, including time limitations, survey perspective limitations, and sample limitations (Huck and Zhang, 2021). The studies conducted during the early stages of the epidemic face time constraints, as participants may have adapted to online distance education behaviors, potentially leading to changes in their learning attitudes and behaviors. Sample limitations refer to small or non-diverse survey sample sizes, which hinder generalizability. The perspective limitation stems from researchers using online surveys, which inherently exclude individuals who may face challenges in filling out online questionnaires.

#### 4.1.1. Procedure

To overcome the aforementioned challenges, this study has devised a well-thought-out research plan. Taking into account the policy implementation background, it is evident that primary and secondary schools in Shenzhen began implementing online education in December 2022 and continued until the end of the semester. The survey took place in March 2023 following the completion of final examinations. The research survey was conducted at the conclusion of the large-scale epidemic prevention and control, ensuring that the participants had already gained online education and learning experience. The sample included students from primary schools, junior high schools, and high schools, representing a diverse range of school types, including private, public, and international overseas schools, thus exhibiting characteristics of sample diversity. To mitigate the perspective limitation caused by technical constraints, the questionnaires were distributed and filled out uniformly by students in the school computer room during their information classes.

#### 4.1.2. Participants

The research participants included students from various types of schools: public, private, and international overseas schools. The surveyed schools encompassed elementary, junior high, and high school levels, excluding students in grades 1–3 due to their cognitive abilities. Data cleaning involved assessing the validity of questionnaires based on response times and answer similarities. In total, this survey included 1,625 valid student questionnaires from 132 classes. The participants consisted of 517 (31.8%) public school students, 888 (54.7%) private school students, and 220 (13.5%) international school students. Among them, there were 735 male students (45.2%) and 890 female students (54.8%). The sample included 791 primary school students (48.7%), 445 junior high school students (27.4%), and 389 senior high school students (23.9%).

### 4.2. Instruments

#### 4.2.1. Learning outcomes: academic achievement

This study measures student learning outcomes using their academic performance in Chinese, Mathematics, and English final exams, such as “Online learning at home made my Chinese performance regress.” A seven-point Likert scale questionnaire with reverse scoring is used to assess students’ perceptions of online education’s impact on their academic performance. SPSS 26.0 is utilized for factor analysis, employing the principal axis factorization method and optimal oblique rotation (kappa = 4). The obtained KMO value is 0.734, and Bartlett’s spherical test is significant. The scale demonstrates a high level of internal consistency reliability with a Cronbach’s α coefficient of 0.866. The academic performance variables are evaluated as saturated models without RMSEA, CFI, TLI, and SRMR values.

#### 4.2.2. Learning participation: online learning engagement

This study assesses students’ Learning Participation using Guo’s (2018) “Students’ Online Learning Engagement Scale” comprising three dimensions: behavioral investment, cognitive investment, and emotional investment. The scale consists of 19 items rated on a Likert seven-point scale, such as “I consistently attend online courses on time,” “I maintain a positive attitude even when facing learning difficulties,” and “I feel a strong sense of accomplishment during online learning.” SPSS 26.0 is used for exploratory factor analysis, and AMOS 24.0 is used for confirmatory factor analysis. The scale demonstrates good reliability (Cronbach’s *α* = 0.947) and validity (χ^2^/df = 10.271, RMSEA = 0.076, CFI = 0.934, TLI = 0.924, SRMR = 0.0376).

#### 4.2.3. Personal characteristic: the Big Five

This study assesses personal characteristics using a measurement scale adapted from Meng et al.’s (2021) “Big Five Personality Scale.” Items include statements such as “I can easily make new friends in my daily study and life,” “I prepare well in advance for my studies,” “I remain calm when faced with study pressure,” “I have a rich imagination in my daily study and life,” and “I show concern when others encounter problems in their daily study and life.” The questionnaire uses a seven-point Likert scale, with 1 indicating “completely disagree” and 7 indicating “completely agree.” Higher scores indicate a higher level of personality fit. SPSS 26.0 is used for exploratory factor analysis, and AMOS 24.0 is used for confirmatory factor analysis. The scale demonstrates good reliability (Cronbach’s *α* = 0.901) and validity (χ^2^/df = 6.180, RMSEA = 0.056, CFI = 0.946, TLI = 0.937, SRMR = 0.0453).

#### 4.2.4. Family environment: family involvement

In this study, student family involvement represents the influence of the family environment on students. The measurement scale is adapted from Wu and Yao’s (2013) “Parents’ Engagement Scale.” It includes four dimensions: home-school communication, home tutoring, participation in decision-making, and life care. The scale consists of 25 items that assess various aspects of family involvement in online education, such as parental consultation with teachers, homework supervision, monitoring academic progress, and providing a reliable internet connection for online learning. The questionnaire employs a seven-point Likert scale. Exploratory factor analysis and confirmatory factor analysis were conducted to establish reliability and validity. The results indicate good reliability (Cronbach’s *α* coefficient = 0.924) and validity (structural validity indices: χ^2^/df = 9.798, RMSEA = 0.074, CFI = 0.890, TLI = 0.878, SRMR = 0.0618), reaching an acceptable level.

#### 4.2.5. School environment: school support

This study examines school support as a representation of the school environment’s impact on students. The measurement scale, developed through focus group deduction, assesses various aspects such as network platform usage, school management and services, teacher teaching, and learning task arrangement. The questionnaire employs a Likert seven-point scoring method. Exploratory factor analysis, using the principal axis factorization method and optimal oblique rotation (kappa = 4), was conducted to establish reliability and validity. The results demonstrate good reliability and validity, as evidenced by the Cronbach’s *α* coefficient (0.944) and confirmatory factor analysis (χ^2^/df = 6.563, RMSEA = 0.059, CFI = 0.994, TLI = 0.990, SRMR = 0.0112).

### 4.3. Data analysis

This study utilizes SPSS 26.0 and AMOS 24.0 for data analysis, employing structural equation modeling to examine the impact mechanism of online learning outcomes. Additionally, the Bootstrap regression path analysis method is employed to test the significance of the model’s mediating effects.

## 5. Results

### 5.1. Descriptive statistics

The descriptive statistics, including the mean, standard deviation, and correlation coefficient, of the main variables in this study are presented in Table 1. The five main variables, namely academic achievement, school support, family involvement, the Big Five, and online learning engagement exhibit positive correlations with each other, aligning with the expectations of this study and providing initial support for the research hypothesis.

**Table 1:** Mean, standard deviation and correlation coefficients.

Variable	Mean	Std.	Academic achievement	School support	Family involvement	The Big Five	Online learning engagement
Academic achievement	4.760	1.683	1.000				
School support	5.422	1.385	0.166***	1.000			
Family involvement	5.477	0.929	0.182***	0.527***	1.000		
The Big Five	5.255	0.913	0.250***	0.527***	0.634***	1.000	
Online learning engagement	5.128	1.081	0.317***	0.605***	0.643***	0.899***	1.000

*p < 0.05, **p < 0.01, ***p < 0.001.

### 5.2. Common method variance

In this study, the Harman single-factor test was employed to assess common method bias (Fuller et al., 2016). Exploratory factor analysis was conducted on all measurement items related to the variables in the model, using principal component analysis without rotation. The first factor explained 29.080% of the total variance, which is below the 40% threshold, indicating that the issue of common method variance is not significant (Xiong et al., 2012).

To further examine the common method bias problem, confirmatory factor analysis was conducted. The model fit indices for this study were less than ideal: χ^2^/df =17.123, RMSEA = 0.100, CFI = 0.478, SRMR = 0.096, NFI = 0.464, TLI = 0.463. These results suggest that there is no substantial common method bias in the data of this study (Malhotra et al., 2006).

### 5.3. Question 1: the SEM model of the online learning ecosystem

**Figure 3:**
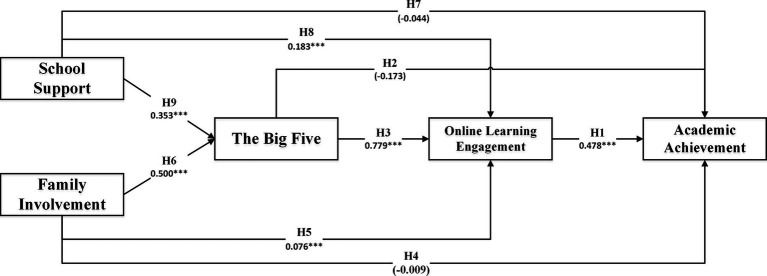
The SEM model of online learning ecosystem. *p < 0.05; **p < 0.01; ***p < 0.001.

To address research question 1, which investigates the interaction between environmental factors, personal characteristics, learning participation, and students’ learning outcomes in the context of online learning, this study constructs an online learning ecosystem mechanism and presents a structural equation model. The model is illustrated in Figure 3.

The structural equation modeling (SEM) analysis of the online education ecosystem yielded favorable model fit indices: χ^2^/df = 4.019, RMSEA = 0.043, CFI = 0.898, SRMR = 0.0902, PNFI = 0.837, TLI = 0.894. The results show a good fit of the model to the data. Table 2 presents the path relationships between variables, confirming the consistency between the assumed theoretical paths and the actual measurement data. The structural model construction and assumptions are ideal, supporting the online learning ecosystem model as a strong theoretical hypothesis model.

**Table 2:** The adaptation results of the online learning ecosystem model.

Statistical testing	Absolute fitness indices			Value-added adaptation indices			Parsimonious fitness indices		
	χ^2^/df	RMSEA	GFI	NFI	IFI	CFI	PNFI	PCFI	PGFI
Parameter	4.019	0.043	0.839	0.869	0.898	0.898	0.837	0.865	0.786

Other parameters: *n* = 1,625; *p* < 0.001; TLI = 0.894; SRMR = 0.0902.

The standardized regression path coefficients of the model are presented in Table 3. Through the joint significance test, it is observed that the coefficients for H2 (the influence of the Big Five on academic achievement), H4 (the influence of family involvement on academic achievement), and H7 (the influence of school support on academic achievement) are not significant. Thus, the assumptions H2, H4, and H7 are not supported by the data. In this study, the mediation effects were tested and analyzed using the Bootstrap method, with 3,000 repeated samples conducted at a 95% confidence interval. The results of the mediation effect test are presented in Table 4. The hypotheses H1, H3, H5, H6, H8, and H9 were supported, confirming the consistency of the model test results with the theoretical framework established in this study.

**Table 3:** The path test of the online learning ecosystem model.

Hypothesis	Std estimate	Standard error	Critical ratio	*p* value	Results
*H1*. Online Learning Engagement →Academic Achievement	0.478	0.171	4.567	***	Supported
*H2*. The Big Five →Academic Achievement	−0.173	0.191	−1.701	0.089	Rejected
*H3*. The Big Five →Online Learning Engagement	0.779	0.059	15.151	***	Supported
*H4*. Family Involvement →Academic Achievement	−0.009	0.056	−0.251	0.802	Rejected
*H5*. Family Involvement →Online Learning Engagement	0.076	0.026	2.759	**	Supported
*H6*. Family Involvement →The Big Five	0.500	0.032	12.551	***	Supported
*H7*. School Support →Academic Achievement	−0.044	0.037	−1.298	0.194	Rejected
*H8*. School Support →Online Learning Engagement	0.183	0.015	7.949	***	Supported
*H9*. School Support →The Big Five	0.353	0.018	11.532	***	Supported

*p < 0.05, **p < 0.01, ***p < 0.001.

**Table 4:** The bootstrap test of the mediation effect.

Path(Std)	Std estimate	Standard error	Lower	Upper	*p* value	Effect ratio
FI → TBF → OLE → AA	0.186	0.052	0.097	0.305	**	42.08%
FI → OLE → AA	0.037	0.022	0.003	0.089	*	8.37%
SS → TBF → OLE → AA	0.131	0.037	0.070	0.221	***	29.64%
SS → OLE → AA	0.087	0.027	0.043	0.150	***	19.68%
The total indirect effect of Family Involvement	0.223	0.060	0.116	0.353	**	50.45%
The total indirect effect of School Support	0.219	0.057	0.114	0.342	**	49.55%
The total effect	0.442	0.113	0.230	0.677	**	

*p < 0.05, **p < 0.01, ***p < 0.001; AA refers to Academic Achievement, SS refers to School Support, FI refers to Family Involvement, TBF refers to The Big Five, OLE refers to Online Learning Engagement.

The study revealed that family Involvement accounted for 50.45% of the total effect, with 42.08% attributed to the indirect pathway of “Family Involvement → The Big Five → Online Learning Engagement → Academic Achievement,” representing 83.41% of the overall effect of family involvement. These findings highlight the significant relationship between family involvement with the Big Five and students’ academic achievement.

Furthermore, school support accounted for 49.55% of the total effect, with 29.64% arising from the pathway of “School Support → The Big Five → Online Learning Engagement → Academic Achievement,” accounting for 60.27% of the effect. Additionally, 19.68% of the effect was attributed to “School Support→ Online Learning Engagement → Academic Achievement,” accounting for 39.73%. These results demonstrate the close association between school support with the Big Five, online learning engagement, and student’s academic achievement.

### 5.4. Question 2: mechanisms of different individual forms and learning participation factors in the online learning ecosystem

#### 5.4.1. School environment: school support

**Figure 4:**
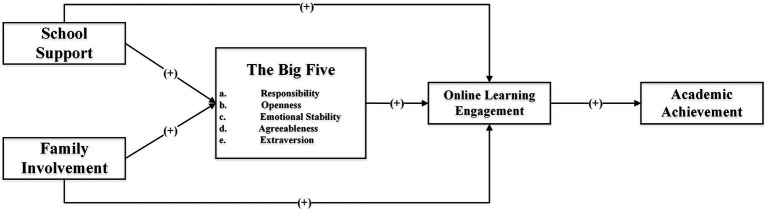
The SEM model of the Big Five in different dimensions.

Since the Big Five plays a crucial mediating role in the relationship between family involvement and school support with students’ academic achievement, this study aims to explore the specific mechanisms of action within this chain mediation effect. Given that the personality traits of different dimensions are subsets of the Big Five, the mediating role of the general concept of the Big Five in the online learning ecosystem can be extrapolated to the personality traits of each dimension. Consequently, this study will exclude the insignificant path effects from the existing online learning ecosystem model, independently examine the five specific personalities within the Big Five, and establish a structural equation model, as depicted in Figure 4.

The SEM fitting indices for the interaction of the Big Five in different dimensions within the online learning ecosystem are as follows: χ^2^/df =4.269, RMSEA = 0.045, CFI = 0.890, SRMR = 0.0961, PNFI = 0.828, TLI = 0.885. These indices indicate a good fit between the model and the data, as shown in Table 5. The model demonstrates that the assumed path relationships align well with the measured data, confirming the soundness of the structural model and its underlying assumptions. Therefore, this model serves as a solid theoretical foundation for the study.

**Table 5:** The model fitting results of the Big Five in different dimensions.

Statistical testing	Absolute fitness indices			Value-added adaptation indices			Parsimonious fitness indices		
	χ^2^/df	RMSEA	GFI	NFI	IFI	CFI	PNFI	PCFI	PGFI
Parameter	4.269	0.045	0.829	0.861	0.890	0.890	0.828	0.856	0.776

Other parameters: *n* = 1,625; *p* < 0.001; TLI = 0.885; SRMR = 0.0961.

The standardized regression path coefficients of the model are presented in Table 6. Through the joint significance test, it is observed that the hypothesis test coefficient of H3e (indicating that extraversion in the Big Five personality contributes to online learning engagement) is negative. This finding contradicts the original hypothesis, suggesting that hypothesis H3e is not established.

**Table 6:** The path test of the Big Five in different dimensions.

Hypothesis	Std estimate	Standard error	Critical ratio	*p* value	Results
*H1*. Online Learning Engagement →Academic Achievement	0.278	0.049	9.559	***	Supported
*H3a*. Responsibility →Online Learning Engagement	0.322	0.023	11.164	***	Supported
*H3b*. Openness →Online Learning Engagement	0.253	0.022	8.739	***	Supported
*H3c*. Emotional Stability →Online Learning Engagement	0.169	0.017	6.178	***	Supported
*H3d*. Agreeableness →Online Learning Engagement	0.112	0.02	4.312	***	Supported
*H3e*. Extraversion →Online Learning Engagement	−0.058	0.017	−2.268	*	Rejected
*H5*. Family Involvement →Online Learning Engagement	0.198	0.05	3.86	***	Supported
*H6a*. Family Involvement →Responsibility	0.535	0.044	14.859	***	Supported
*H6b*. Family Involvement →Openness	0.588	0.048	16.138	***	Supported
*H6c*. Family Involvement →Emotional Stability	0.553	0.055	15.717	***	Supported
*H6d*. Family Involvement →Agreeableness	0.547	0.046	15.512	***	Supported
*H6e*. Family Involvement →Extraversion	0.506	0.052	14.343	***	Supported
*H8*. School Support →Online Learning Engagement	0.27	0.015	11.514	***	Supported
*H9a*. School Support →Responsibility	0.182	0.021	7.094	***	Supported
*H9b*. School Support →Openness	0.159	0.022	6.49	***	Supported
*H9c*. School Support →Emotional Stability	0.188	0.026	7.527	***	Supported
*H9d*. School Support →Agreeableness	0.121	0.021	4.9	***	Supported
*H9e*. School Support →Extraversion	0.148	0.026	5.74	***	Supported

*p < 0.05, **p < 0.01, ***p < 0.001.

In this study, the same Bootstrap procedure as described above was employed to test and analyze the mediating effect, and the specific results are presented in Table 7.

**Table 7:** The bootstrap test of the mediation effect.

Path (Std)	Std estimate	Standard error	Lower	Upper	*p* value	Effect ratio
FI → Respo →OLE → AA	0.048	0.008	0.034	0.068	***	36.36%
FI → Open →OLE → AA	0.041	0.008	0.028	0.059	***	31.06%
FI → Emo → OLE → AA	0.026	0.006	0.016	0.039	***	19.70%
FI → Agree→ OLE → AA	0.017	0.006	0.007	0.028	**	12.88%
SS → Respo→ OLE → AA	0.016	0.005	0.009	0.028	***	40.00%
SS → Open→ OLE → AA	0.011	0.004	0.005	0.021	***	27.50%
SS → Emo → OLE → AA	0.009	0.003	0.004	0.017	***	22.50%
SS → Agree→ OLE → AA	0.004	0.002	0.001	0.009	**	10.00%
FI → TBF → OLE → AA	0.132	0.017	0.102	0.173	***	
SS → TBF → OLE → AA	0.040	0.010	0.023	0.063	***	

*p < 0.05, **p < 0.01, ***p < 0.001; FI refers to Family Involvement, AA refers to Academic Achievement, TBF refers to The Big Five, OLE refers to Online Learning Engagement, Respo refers to Responsibility, Open refers to Openness, Emo refers to Emotional Stability, Agree refers to Agreeableness.

The study found that the effects of agreeableness, emotional stability, openness, and responsibility in the chain mediation of family investment and the specific personality traits of the Big Five increased sequentially.

Similarly, in the chain mediation of school investment and the Big Five personality traits, the effects of agreeableness, emotional stability, openness, and responsibility also increased sequentially.

#### 5.4.2. Different forms of online learning engagement

**Figure 5:**
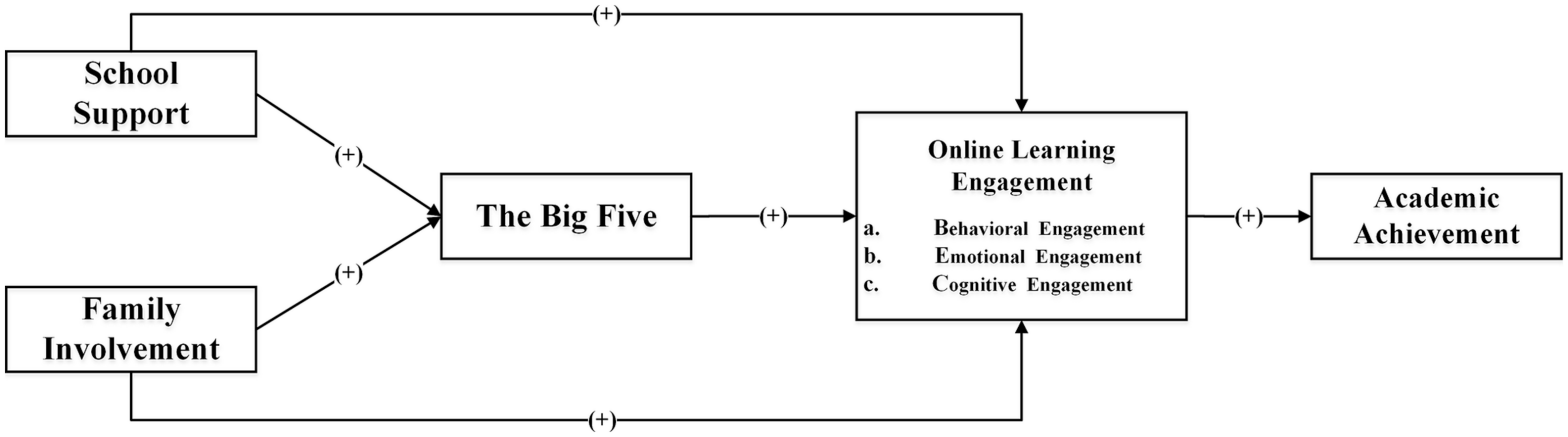
The SEM model of Online Learning Engagement in different dimensions.

In the context of online learning, school support plays a crucial role in influencing students’ academic achievement through its mediation effect on online learning input. This study aims to examine the specific mechanism of different dimensions of online learning engagement in this chain mediation effect. As various forms of learning engagement are subsets of online learning engagement, the mediating role of the general concept of online learning engagement in the online learning ecosystem can be applied to each form of learning engagement. Thus, insignificant path effects are omitted in the existing online learning ecosystem model, and a separate structural equation model is developed to represent the three methods of online learning engagement, as depicted in Figure 5.

The SEM model for the interaction of different forms of online learning engagement in the online learning ecosystem demonstrates favorable model fit indices: χ^2^/df =4.042, RMSEA = 0.043, CFI = 0.897, SRMR = 0.0904, PNFI = 0.836, TLI = 0.893. Table 8 provides detailed information on the model fit results. The findings indicate that the assumed path relationship aligns well with the measured data, confirming the appropriateness of the structural model construction and assumptions. Thus, the model for the interaction of different forms of online learning engagement in the online learning ecosystem aligns with sound theoretical assumptions.

**Table 8:** The model-fitting results of different Online Learning Engagement form.

Statistical testing	Absolute fitness indices			Value-added adaptation indices			Parsimonious fitness indices		
	χ^2^/df	RMSEA	GFI	NFI	IFI	CFI	PNFI	PCFI	PGFI
Parameter	4.042	0.043	0.838	0.868	0.897	0.897	0.836	0.864	0.784

Other parameters: *n* = 1,625; *p* < 0.001; TLI = 0.893; SRMR = 0.0904.

The results of the standardized regression path coefficients of the model are presented in Table 9. Based on the joint significance test, it was found that the hypothesis test coefficient of H1c (cognitive engagement in online learning engagement is beneficial to academic achievement) is not statistically significant, indicating that hypothesis H1c is not supported by the data.

**Table 9:** The path test of Online Learning Engagement in different dimensions.

Hypothesis	Std estimate	Standard error	Critical ratio	*p* value	Results
*H1a*. Behavioral Engagement →Academic Achievement	0.229	0.067	4.891	***	Supported
*H1b*. Emotional Engagement →Academic Achievement	0.125	0.047	3.251	**	Supported
*H1c*. Cognitive Engagement →Academic Achievement	−0.033	0.064	−0.703	0.482	Rejected
*H8a*. School Support →Behavioral Engagement	0.057	0.018	2.467	*	Supported
*H8b*. School Support →Emotional Engagement	0.213	0.023	8.225	***	Supported
*H8c*. School Support →Cognitive Engagement	0.064	0.019	2.693	**	Supported

*p < 0.05, **p < 0.01, ***p < 0.001.

In this study, the mediation effects were tested and analyzed using the Bootstrap method, with 3,000 repeated samples conducted at a 90% confidence interval, and the specific results are presented in Table 10.

**Table 10:** The bootstrap test of the mediation effect.

Path (Std)	Std estimate	Standard error	Lower	Upper	*p* value	Effect ratio
SS → Behavioral →AA	0.013	0.009	0.001	0.032	0.063	32.50%
FI → Emotional→ AA	0.027	0.010	0.012	0.045	*	67.50%
SS → OLE → AA	0.040	0.014	0.019	0.067	*	

*p < 0.05, **p < 0.01, ***p < 0.001; SS refers to School Support, AA refers to Academic Achievement, OLE refers to Online Learning Engagement, Behavioral refers to Behavioral engagement, Emotional refers to Emotional engagement.

The study revealed that in the chain mediation effect of school support and different forms of online learning engagement on academic achievement, the proportion of behavioral engagement and emotional engagement effect increased sequentially. Specifically, 32.50% of the effect can be attributed to the intermediary path of “School Support → Behavioral Engagement → Academic Achievement,” while 67.50% of the effect can be attributed to the intermediary path of “School Support→ Emotional Engagement → Academic Achievement.”

## 6. Discussion

Our investigation, rooted in ecosystem theory, has yielded significant insights into the intricate web of factors influencing learning outcomes in K-12 online education. By aligning our findings with the tenets of the theoretical framework and existing literature, we can unravel the nuanced dynamics at play.

### 6.1. Direct and indirect influences on learning outcomes

As proposed by our model, learning outcomes in the online learning ecosystem are shaped by direct and indirect influences. Online learning engagement emerges as a potent direct predictor of learning outcomes. This underscores the pivotal role of active participation and engagement in maintaining students’ enthusiasm and commitment within the flexible realm of online education. In contrast to the traditional classroom environment, the online learning setting is distinctive, offering greater freedom and enhanced flexibility. As a result, students participating in online learning need to possess heightened self-management and self-control abilities (Wang et al., 2013), a contention further substantiated by the findings of our study.

Furthermore, our findings highlight the intricate indirect pathways through which individual characteristics and environmental factors influence learning outcomes. The Big Five personality traits and living environment exert their impact not through direct channels, but via the mediating role of “online learning participation engagement.” This resonates with the personalized nature of online learning, which values individual differences and caters to specific student needs (Theobald et al., 2018; Costa et al., 2020).

In the investigation of both direct and indirect factors influencing learning outcomes in online education, the perspective of ecosystem theory (Bronfenbrenner and Morris, 2007) offers a comprehensive multidimensional lens for our research. By integrating individual and environmental factors into an integrated framework guided by this theory, we unveil the intricate interplay among these diverse dimensions. While various previous studies have delved into influencing factors, many have primarily focused on single-dimensional exploration rather than embracing a multi-dimensional approach. The outcomes yielded by our model not only confirm the existence of the multidimensional framework we constructed, but also validate the applicability of ecosystem theory within the domain of online education.

### 6.2. Family and school environments

In our study, the examination of family and school environments offers novel insights into the dual influences shaping students’ learning outcomes in the online realm. Our findings demonstrate that both the family environment and the school environment have similar effects on students’ learning outcomes during online learning. Remarkably, the influence of the home environment on learning outcomes even marginally surpasses that of the school environment. This phenomenon may stem from the fact that in traditional educational settings, schools often leverage tangible resources such as infrastructure (Alexander and Eckland, 1977), human capital in the form of teachers (Aaronson et al., 2007), and peer effects (Rumberger and Palardy, 2005). These mechanisms synergistically contribute to students’ educational attainment. However, the distinct learning approach of home-based online education underscores the significance of electronic resources, necessitates the adaptation of teachers from offline to online modes, reduces face-to-face interactions, and thereby transforms the traditional dynamics of school education. Consequently, the potency of family-related factors in influencing children’s learning and overall growth is amplified. This outcome aligns with the conclusions drawn by Liu et al. (2022) that parents possess a pivotal role in their children’s online learning journey. Moreover, this finding concurs with the research outcomes of Tao and Xu (2022), who underscore that students often require the combined support of teachers and parents to navigate the self-regulation process inherent to online learning.

### 6.3. The role of Big Five personality traits

The nuanced relationship between the Big Five personality traits and learning outcomes offers intriguing insights. This study examined the mediating effect of each dimension of the Big Five personality traits and identified conscientiousness, openness, and emotional stability as having a primarily significant positive impact on the established online learning ecosystem model. These findings show slight deviations from research outcomes in traditional education (Poropat, 2009), where conscientiousness, openness, and agreeableness were identified to exert significant positive effects on learning outcomes within conventional school environments. In contrast, our study reveals that the impact mechanism of online education learning outcomes places an emphasis on emotional stability. In fact, emotional stability has been confirmed to play a crucial role in online education (Ninggal et al., 2020). In comparison to the conventional learning setting, the heightened flexibility of the online learning process introduces additional challenges for students’ learning. Participants engaged in online learning are required to adeptly manage stress, and those possessing high emotional stability are more likely to maintain a composed emotional state, thus enhancing their stress management abilities (Gagani et al., 2021).

### 6.4. Emotional engagement in online learning

Our findings underscore the substantial role played by emotional engagement within the online learning ecosystem. This prominence can be attributed to the virtualized learning environment that students encounter in the realm of online education. In stark contrast to traditional education, the avenues for face-to-face communication and interaction are constrained in online learning, thereby accentuating the pivotal role of emotional engagement for students. This finding aligns with the research findings of Yu et al. (2020), who demonstrated that by mediating the dynamics among student-teacher interaction, student-content interaction, and sustained learning commitment within the online learning environment, emotional engagement emerges as a critical factor in facilitating effective student engagement. Therefore, schools can enhance students’ emotional engagement by fostering their learning enthusiasm, stimulating their interest in learning, and creating a positive learning atmosphere, ultimately leading to improved learning outcomes (Zhen et al., 2017).

In conclusion, our study not only contributes empirical insights but also aligns these findings with the ecosystem theory and existing literature. The interplay between direct and indirect influences, the impacts of family and school environments, the role of personality traits, and the significance of emotional engagement collectively underscore the intricate dynamics that govern learning outcomes in the evolving landscape of K-12 online education.

## 7. Contributions and implications

### 7.1. Theoretical contributions

The study unfolds several noteworthy theoretical contributions that emanate directly from the insights garnered through rigorous analysis of the data. These contributions are as follows:

#### 7.1.1. Bridging the gap in K-12 online learning research

This study holds paramount significance as the first systematic exploration into the impact mechanism of K-12 online learning. The prevailing discourse on online learning predominantly centers around higher education, inadvertently sidelining the pivotal role of K-12 students in the post-epidemic era. Our research rectifies this oversight by delving into the impact dynamics specifically within the K-12 context, offering insights that are inherently relevant to the core of online learning’s evolution. To some extent, our exploration, which might not be categorized strictly as a theoretical contribution, serves as a crucial foundation for understanding the dynamics involved in K-12 online education.

#### 7.1.2. Developing a holistic conceptual model based on ecosystem theory

This study employs the ecosystem theory to comprehensively scrutinize the impact mechanisms of learning outcomes in online education. Unlike conventional studies that often focus on singular factors within specific categories, our model, constructed on the foundation of ecosystem theory, seamlessly integrates the learning process, personal characteristics, and environmental factors. This comprehensive approach addresses a gap in the understanding of impact mechanisms on learning outcomes in online education. Crucially, the empirical validation of this framework reinforces its potency in explicating learning outcomes within the K-12 online learning milieu. It is important to underscore that our model represents one plausible interpretation, and we acknowledge the potential for other researchers to propose new and refined models in the future. The value of our model lies not only in its current application but also in its potential to inspire further advancements in the exploration of online learning outcomes.

#### 7.1.3. Offering novel insights into learning outcome mechanism

Our investigation into the relationship between individual factors, learning participation process factors, and environmental factors has provided insights into a previously unknown mechanism of K-12 students’ learning outcomes in the context of online learning. On one hand, our study builds upon existing qualitative research by quantitatively analyzing the combined effects of the family and school environments, demonstrating that they have almost equal impacts on online academic achievement. On the other hand, our findings highlight the disparities between traditional offline learning and online learning. Specifically, conscientiousness, openness, and emotional stability among Big Five Personality traits significantly and positively influence the established online learning ecosystem model, while emotional engagement from school support in online learning has the most significant impact.

In essence, our theoretical contributions are firmly rooted in the empirically-derived insights that our study provides. By bridging the gap in K-12 online learning research, proposing a comprehensive framework, and revealing the nuances of learning outcome mechanisms, our study enriches the theoretical landscape. Importantly, our model is a stepping stone, inviting further exploration by future researchers.

### 7.2. Practical implications

This study offers valuable strategic guidance to families and schools in navigating future learning scenarios characterized by uncertain combinations of on-campus learning, online learning, and blended learning.

Family involvement in these variables should prioritize the cultivation of their children’s responsible and open personality traits. In the context of online learning, family involvement primarily influences students’ academic achievement through two intermediary pathways: their responsibility and openness personality traits. It is important to note that children’s personality traits are closely linked to the nurturing behavior of parents (Muris, 2006). When the family support environment remains stable, individual personality traits tend to remain stable as well. However, in the face of changing environments, personality traits have the tendency to shift in the same direction (Branje et al., 2004). Therefore, establishing a family environment that fosters the development of responsible and open personality traits becomes an effective approach to harnessing the influence of the family context on learning outcomes.

When schools support online learning, it is essential for them to enhance the quality of teachers and prioritize emotional education. Within the context of online learning, the influence of school investment on academic achievement is closely associated with two mediating paths: students’ responsibility and openness personality traits, as well as students’ emotional engagement. Teachers play a significant role in shaping students’ personalities (Ulug et al., 2011). Therefore, schools should prioritize the training and professional development of teachers. Some key areas of training include online learning tools, teaching strategies, and student psychological and emotional support. This will enable teachers to effectively guide students’ personal growth and enhance academic achievement. Additionally, schools should focus on fostering students’ emotional engagement. This can be achieved through encouraging innovative teaching methods, creating a positive learning environment, and providing opportunities for students to experience positive emotions while learning and socializing. By doing so, schools can effectively stimulate students’ enthusiasm and passion for learning.

### 7.3. Limitations and future directions

First and foremost, it is important to note that the results of this study are solely derived from a self-reported questionnaire survey completed by students. Consequently, there may exist a self-leniency tendency that could impact the determination of causal relationships. Therefore, future research endeavors should aim to incorporate non-self-reported data and collect information from multiple sources to measure the same variables. This approach would allow for a comprehensive examination by combining subjective and objective data, thereby enhancing the effectiveness of the questionnaire method and achieving a more robust analysis of the research subject.

Secondly, it is important to note that this study conducted a questionnaire survey in April 2023 to investigate the academic impact of online learning in December 2022. Although the final exam for the fall semester of 2022 was rescheduled to the spring of 2023, the survey was conducted immediately after the release of the exam results. However, it is worth considering that there was a Spring Festival holiday between the final exam and online learning, and it cannot be ruled out the possibility that students’ learning behavior during the holiday may have influenced the research findings. Therefore, future research in the field of online education for basic education should take measures to eliminate the potential interference of this factor and strive to maintain the continuity of online learning and learning assessment.

Finally, it is crucial to acknowledge the limitations of this study in terms of research design. Because the sample of this study includes elementary school students, junior high school students, and high school students from different types of schools, the research results do possess a certain degree of representativeness and wide applicability. However, due to the limited space of the article, the focus remains on analyzing the impact mechanism of online education on learning outcomes. As a result, a specific comparative analysis across school stages and types was not conducted. The school stage and type may impact online learning adaptability, learning habits, and outcomes. Differences in the learning environment and motivation across school stages and types may influence students’ learning outcomes. In the future, it would be beneficial to conduct further studies to explore these differences in more detail.

## Data availability statement

The raw data supporting the conclusions of this article will be made available by the authors, without undue reservation.

## Ethics statement

Ethical approval was not required for the study involving human samples in accordance with the local legislation and institutional requirements. Written informed consent for participation in this study was provided by the participants’ legal guardians/next of kin.

## Author contributions

PW: project administration, writing – original draft, methodology, and data analysis. FW: supervision, funding acquisition, and editing. ZL: conceptualization and questionnaire survey. All authors contributed equally to the article and approved the submitted version.
